# A systematic review of the quality and scope of economic evaluations in child oral health research

**DOI:** 10.1186/s12903-019-0825-2

**Published:** 2019-07-01

**Authors:** H. J. Rogers, H. D. Rodd, J. H. Vermaire, K. Stevens, R. Knapp, S. El Yousfi, Z. Marshman

**Affiliations:** 1 Unit of Oral Health, Dentistry and Society, School of Clinical Dentistry, University of Sheffield, Sheffield, UK; 2 Division of Child Health, TNO Institute for Applied Sciences, Leiden, The Netherlands; 3 Health Economics and Decision Science, School of Health and Related Research, University of Sheffield, Sheffield, UK

**Keywords:** Paediatric, Oral health, Health economics, Cost-effectiveness, Dentistry

## Abstract

**Background:**

Economic evaluations provide policy makers with information to facilitate efficient resource allocation. To date, the quality and scope of economic evaluations in the field of child oral health has not been evaluated. Furthermore, whilst the involvement of children in research has been actively encouraged in recent years, the success of this movement in dental health economics has not yet been explored. This review aimed to determine the quality and scope of published economic evaluations applied to children’s oral health and to consider the extent of children’s involvement.

**Methods:**

The following databases were searched: CINAHL, Cochrane Library, Econlit, EThOS, MEDLINE, NHS EED, OpenGrey, Scopus, Web of Science. Full economic evaluations, relating to any aspect of child oral health, published after 1997 were included and appraised against the Drummond checklist and the Consolidated Health Economic Evaluation Reporting Standards by a team of four calibrated reviewers. Data were also extracted regarding children’s involvement and the outcome measures used.

**Results:**

Two thousand seven hundred fifteen studies were identified, of which 46 met the inclusion criteria. The majority (*n* = 38, 82%) were cost-effectiveness studies, with most focusing on the prevention or management of dental caries (*n* = 42, 91%). One study quantified outcomes in Quality Adjusted Life Years (QALYs), and one study utilised a child-reported outcome measure.

The mean percentage of applicable Drummond checklist criteria met by the studies in this review was 48% (median = 50%, range = 0–100%) with key methodological weaknesses noted in relation to discounting of costs and outcomes. The mean percentage of applicable CHEERS criteria met by each study was 77% (median = 83%, range = 33–100%), with limited reporting of conflicts of interest. Children’s engagement was largely overlooked.

**Conclusions:**

There is a paucity of high-quality economic evaluations in the field of child oral health. This deficiency could be addressed through the endorsement of standardised economic evaluation guidelines by dental journals. The development of a child-centred utility measure for use in paediatric oral health would enable researchers to quantify outcomes in terms of quality adjusted life years (QALYs) whilst promoting child-centred research.

## Background

There are a number of current problems facing children’s oral health globally, the first and foremost being dental caries. A recent systematic review reported 9% of children worldwide have untreated caries, highlighting it as a major international public health problem [1]. In the United Kingdom (UK), approximately 57,485 children aged up to 19-years were admitted to hospital in 2015–2016 with a diagnosis of dental caries, making it the most common reason for children to require an admission with an estimated cost of £39 million to the National Health Service (NHS) [2, 3]. Similarly, a ten-year study of dental admission patterns from 2000 to 2009 in Western Australian children aged 14 years and younger identified 43,937 children who had been hospitalised for an oral health-related condition [4].

Whilst dental caries is the most prevalent dental problem to affect children, other common childhood dental conditions also present considerable financial burden to both families and healthcare providers. One third of all British preschool children suffer a traumatic dental injury involving the primary dentition whilst one in four school children sustain dental injury to the permanent dentition [5]. Molar incisor hypomineralisation (MIH), cited as affecting between 10 to 20% of children globally, is being increasing viewed as a public health concern [6, 7]. Furthermore, the Child Dental Health Survey in England, Wales and Northern Ireland 2013 found that 9 and 18% of 12- and 15-year olds respectively were undergoing orthodontic treatment, utilising a considerable proportion of the NHS dental budget [8].

For each dental condition, a range of interventions can be employed with differing levels of clinical effectiveness and costs. Economic evaluations seek to compare both the cost and benefits of two or more healthcare interventions to provide clinicians and policy makers with the information required to utilise resources in the most efficient way. In the UK, the National Institute for Health and Care Excellence (NICE) uses a combination of clinical and economic evidence to develop guidance and recommendations on the use of new and existing interventions within the remit of the National Health Service (NHS). A similar approach is utilised by decision-makers in other countries around the world, including PHARMAC in New Zealand [9].

As economic evaluations are often used to inform decision-makers, it is essential that they are of robust scientific quality. A previous systematic review of economic evaluations relating to dentistry identified a number of methodological flaws [10]. A more recent review also highlighted deficiencies in the reporting of economic evaluations of oral health interventions [11]. However, neither of these systematic reviews explored both the methodological and reporting quality of the economic evaluations nor had a specific focus on children’s oral health research.

There now exists persuasive evidence that children and young people are able to report on their own health and should be involved in healthcare decisions [12]. Health researchers are therefore encouraged to consider children as active participants. Whilst children’s engagement is becoming increasingly evident in some areas of child oral health research, little is known about their contribution to the field of economic evaluation [13, 14].

The aim of this systematic review, therefore, was to examine both the quality and scope of economic evaluations in the field of child oral health research. The following specific objectives were set:

● To describe frequency and trends in the publication of economic evaluations in child oral health research

● To explore the extent to which children have been involved in economic evaluations of child oral health

● To examine the quality of published economic evaluations in child oral health research using two quality assessment tools specifically developed for appraisal of economic evaluations.

## Methods

A search strategy was developed iteratively, combining search terms relating to the key concepts with adaptations of the validated University of York’s Centre for Reviews and Dissemination (CRD) economic evaluation search filter for the databases MEDLINE, EMBASE and CINAHL. The search filters were then modified further and used to search the following databases: NHS Economic Evaluation Database (CRD York), Web of Science, Scopus, the Cochrane Library and Econlit. Each search covered the period from commencement of each database system until the initiation of the systematic review (January, 2017).

Bibliographic information from identified studies was examined for further applicable titles. Efforts were made to identify relevant unpublished ‘grey’ literature, theses and conference proceedings through appropriate websites and the databases OpenGrey and EThOS.

Search results were de-duplicated and organised using EndNote™ X8.1. Potentially relevant titles and abstracts were screened against the inclusion and exclusion criteria below by one reviewer (HJR).

### Inclusion criteria

 ● Studies involving children aged 18 years old and under

● Studies including a full economic evaluation in the field of child oral health

*(It should be noted that although the reviewing team had some concerns that cost-minimisation studies may not be universally considered as full economic evaluations, for completeness they were included in this review)*

● Studies published after 1997

(*After discussion between all reviewers, it was agreed that studies published in and prior to 1997 should be excluded from this review. This was due to the limited guidance available to researchers before the publication and wider dissemination of the Drummond checklist* [15] *which was to be employed for this current review)*

### Exclusion criteria

 ● Studies including participants over 18 years of age

 ● Decision models extending past 18 years of age

*(For the purposes of this systematic review, the research team decided to exclude studies involving decision models that extended into adulthood, or over a lifetime, in order to focus on the benefits from interventions gained solely during the childhood period)*

● Studies not in the field of oral health

● Studies published in and prior to 1997

Full texts were retrieved for all titles appearing to meet these criteria, with no language restrictions. Two reviewers (HJR and EV) then assessed the full texts against the inclusion and exclusion criteria independently, with any disagreement resolved by consensus. Input from a third reviewer (KS) was sought where required, and translators were used when necessary.

Additional details including type of economic evaluation, publication year, outcome reporting and outcome measures were extrapolated to a Microsoft Excel spreadsheet by two members of the research team (RK and SE), following a data extraction training exercise. When extracting data regarding outcome reporting, one or more of the following options were selected:

1. Clinician-reported (e.g. DMFT, IOTN)
2. Parent-reported (e.g. P-CPQ)
3. Child-reported (e.g. CARIES-QC, CHU9D)
4. Combination
5. Not applicable (e.g. for studies using data from multiple studies/model-based studies)

These options were adapted from two previous systematic reviews of oral health-related literature to establish the involvement of children [16, 17].

### Evaluation tools

There are numerous guidelines available to support researchers and economists in producing high quality economic evaluations with the most widely used being the aforementioned Drummond 10-item, 13-criteria checklist [15]. This is a simplified version of the more detailed 35-item Drummond version, providing comprehensive guidance on the methodological conduct of an economic evaluation. It is recommended in the Cochrane Handbook for Systematic Reviews of Interventions [18, 19]. The Drummond checklist was used in this systematic review to assess the methodological quality of the included studies, in conjunction with the novel Consolidated Health Economic Evaluation Reporting Standards (CHEERS) checklist [20].

The CHEERS checklist was developed by the International Society for Pharmacoeconomics and Outcomes Research (ISPOR) Health Economic Evaluation Publication Guidelines Good Reporting Practices Task Force, in response to an acknowledged need for consolidated, updated and user-friendly reporting guidelines. Published in 2013, these standards provide a 24-item checklist, with accompanying recommendations and examples, with the overall aim of ensuring more consistent and transparent reporting in this field. The CHEERS checklist has been used in several published systematic reviews of economic evaluations assessing healthcare interventions, including one in the field of oral health interventions which was published during the course of this systematic review [11, 21].

Two reviewers assessed the methodological quality of each study against the Drummond checklist, whilst two further reviewers assessed reporting quality using the CHEERS checklist. A score of 0, 1 or 2 was allocated by the reviewers for each criterion as follows:

Score 0: Criterion not met

Score 1: Criterion met

Score 2: Criterion not applicable.

A calibration exercise was conducted with all reviewers prior to commencement of the quality appraisal to enable familiarisation with both checklists, to gain consistency in scoring. Resolution of disagreement was achieved through discussion and involvement of a third reviewer to reach a consensus. Data extraction and quality appraisal were undertaken by one reviewer (HJR) with an appropriate translator for those included studies published in languages other than English.

Simple descriptive statistics were undertaken on the extracted data and quality appraisal results using IBM® SPSS® Statistics v23, alongside a narrative synthesis.

## Results

**Fig. 1:**
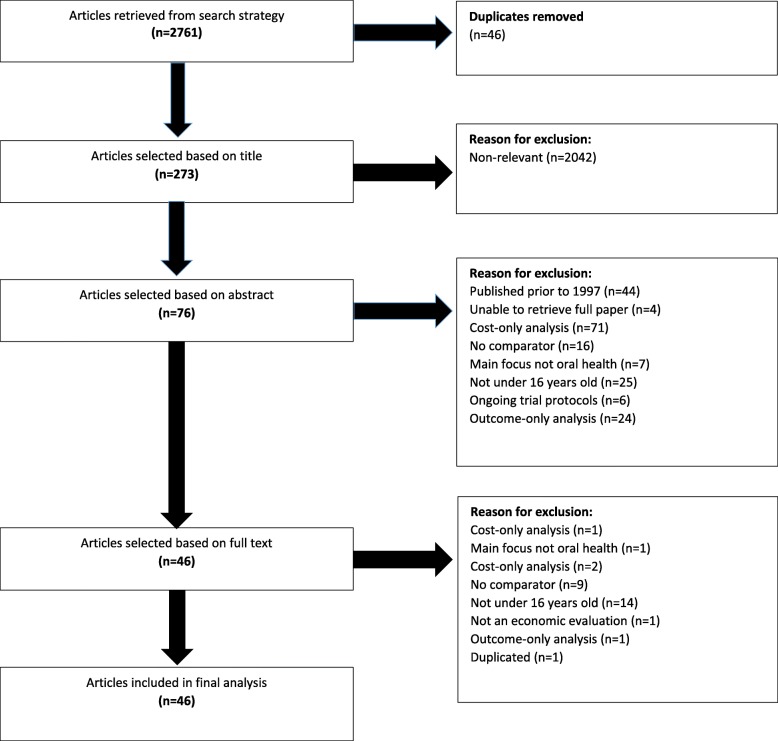
Flowchart displaying search process

The search process, depicted in Fig. 1, was first conducted on 17th January, 2017. The search was then repeated on 5th June, 2017 to identify further, more recent publications that could meet the search criteria. This identified a further four studies. However, three failed to meet the inclusion criteria, and one had already been identified in the initial search. A summary of the included studies [22–67] can be found in Table 1.

**Table 1:** Characteristics of studies included in systematic review

First Author	Title	Year of publication	Country	Type of EE	Condition studied	Measure of effect used	Outcome reporting
*Alkhadra, T.* [22]	Cost -effectiveness of a pit and fissure sealants program in a school-based setting in Saudi Arabia	2004	Saudi Arabia	CEA	Caries	Caries vs no caries	Clinician
*Atkins, C.* [23]	Cost-effectiveness of preventing dental caries and full mouth dental reconstructions among Alaska Native children in the Yukon–Kuskokwim delta region of Alaska	2016	USA	CEA	Caries	Number of caries prevented	Clinician
*Bergström, E.* [24]	Caries and costs: An evaluation of a school-based fluoride varnish programme for adolescents in a Swedish region	2016	Sweden	CMA	Caries	DFT,DFSa,DeSa	Clinician
*Bertrand, É.* [25]	Cost-effectiveness simulation of a universal publicly funded sealants application program	2011	Canada	CEA	Caries	Number of children without decay on first permanent molars	Clinician
*Bhuridej, P.* [26]	Four-year cost-utility analyses of sealed and nonsealed first permanent molars in Iowa Medicaid-enrolled children	2007	USA	CUA	Caries	QATY	Clinician
*Chi, D.* [27]	Cost-Effectiveness of Pit-and-Fissure Sealants on Primary Molars in Medicaid-Enrolled Children	2014	USA	CEA	Caries	Number of teeth restored or extracted	Clinician
*Davies, G.* [28]	An assessment of the cost effectiveness of a postal toothpaste programme to prevent caries among five-year-old children in the North West of England	2003	UK	CEA	Caries	dmft	Clinician
*Frazão, P.* [29]	(Cost-effectiveness of conventional and modified supervised toothbrushing in preventing caries in permanent molars among 5-year-old children)	2012	Brazil	CEA	Caries	Incidence density	Clinician
*Goldman, A.* [30]	Methods and preliminary findings of a cost-effectiveness study of glass-ionomer-based and composite resin sealant materials after 2 yr	2014	China	CEA	Caries	dmft,DMFT	Clinician
*Goldman, A.* [31]	Cost-effectiveness, in a randomized trial, of glass-ionomer-based and resin sealant materials after 4 yr	2016	China	CEA	Caries	dmft,DMFT	Clinician
*Griffin, S.* [32]	Comparing the costs of three sealant delivery strategies	2002	USA	CEA	Caries	Annual first permanent molar occlusal surface caries increment	Clinician
*Hichens, L.* [33]	Cost-effectiveness and patient satisfaction: Hawley and vacuum-formed retainers	2007	UK	CEA	Malocclusion	Little’s irregularity Index and patient satisfaction questionnaire	Child and clinician
*Hietasalo, P.* [34]	Cost-effectiveness of an experimental caries-control regimen in a 3.4-yr randomized clinical trial among 11–12-yr-old Finnish schoolchildren	2009	Finland	CEA	Caries	DMFS	Clinician
*Hirsch, G.* [35]	A simulation model for designing effective interventions in early childhood caries	2012	USA	CEA	Caries	DFT	N/A
*Holland, T.* [36]	The effectiveness and cost of two fluoride program for children	2001	Ireland	CEA	Caries	DMFT	Clinician
*Jokela, J.* [37]	Economic evaluation of a risk-based caries prevention program in preschool children	2003	Finland	CEA	Caries	Caries developed, time spent on treatment	Clinician
*Kaakko, T.* [38]	An ABCD program to increase access to dental care for children enrolled in Medicaid in a rural county	2002	USA	CEA	Caries	Rate of utilisation and dmft	Clinician
*Koh, R.* [39]	Relative cost-effectiveness of home visits and telephone contacts in preventing early childhood caries	2015	Australia	CEA + CUA	Caries	QALYs	N/A
*Kowash, M.* [40]	Cost-effectiveness of a long-term dental health education program for the prevention of early childhood caries	2006	UK	CEA + CBA	Caries	dmft/s	Clinician
*Leskinen, K.* [41]	Practice-based study of the cost-effectiveness of fissure sealants in Finland	2008	Finland	CEA	Caries	Surface-specific filling increments of permanent first molars and incisors	Clinician
*Marino, R.* [42]	Modeling an economic evaluation of a salt fluoridation program in Peru	2011	Peru	CEA	Caries	DMFT	Clinician
*Mariño, R.* [43]	Cost-effectiveness models for dental caries prevention programmes among Chilean school children	2012	Chile	CEA	Caries	DMFT	Clinician
*Mariño, R.* [44]	The cost-effectiveness of adding fluorides to milk-products distributed by the National Food Supplement Programme (PNAC) in rural areas of Chile	2007	Chile	CEA	Caries	dmft	Clinician
*Morgan, M.* [45]	Economic evaluation of a pit and fissure dental sealant and fluoride mouthrinsing program in two nonfluoridated regions of Victoria, Australia	1998	Australia	CEA	Caries	DMFS	Clinician
*Neidell, M.* [46]	Cost-Effectiveness Analysis of Dental Sealants versus Fluoride Varnish in a School-Based Setting	2016	USA	CEA	Caries	% caries reduction	Clinician
*Ney, J. P.* [47]	Economic modeling of sealing primary molars using a “value of information” approach	2014	USA	CEA	Caries	Restorations or extractions averted	Clinician
*Oscarson, N.* [48]	Cost-effectiveness of different caries preventive measures in a high-risk population of Swedish adolescents	2003	Sweden	CEA	Caries	DMFS	Clinician
*Ouyang, W.* [49]	Cost -effectiveness analysis of dental sealant using econometric modeling	2009	USA	CEA	Caries	Presence of caries	Clinician
*Petrén, S.* [50]	Early correction of posterior crossbite-a cost-minimization analysis	2013	Sweden	CMA	Malocclusion	Success rate of crossbite correction and degree of maxillary expansion in mm	Clinician
*Pukallus, M.* [51]	Cost-effectiveness of a telephone-delivered education programme to prevent early childhood caries in a disadvantaged area: a cohort study	2013	Australia	CEA	Caries	Number of carious teeth	Clinician
*Quiñonez, R.* [52]	Assessing cost-effectiveness of sealant placement in children	2005	USA	CEA	Caries	Cavity-free months	Clinician
*Quinonez, R.* [53]	Simulating cost-effectiveness of fluoride varnish during well-child visits for Medicaid-enrolled children	2006	USA	CEA	Caries	Cavity-free months	Clinician
*Ramos-Gomez, F.* [54]	Cost-effectiveness model for prevention of early childhood caries	1999	USA	CEA	Caries	dmfs	Clinician
*Sakuma, S.* [55]	Economic Evaluation of a School-based Combined Program with a Targeted Pit and Fissure Sealant and Fluoride Mouth Rinse in Japan	2010	Japan	CEA	Caries	DFT	Clinician
*Samnaliev, M.* [56]	Cost-effectiveness of a disease management program for early childhood caries	2015	USA	CEA	Caries	Hospital based visits for restorative treatment or extractions	Clinician
*Sköld, U.* [57]	Cost-analysis of school-based fluoride varnish and fluoride rinsing programs	2008	Sweden	CEA	Caries	Prevented fillings	Clinician
*Stearns, S.* [58]	Cost-effectiveness of preventive oral health care in medical offices for young medicaid enrollees	2012	USA	CEA	Caries	Visits for dental treatment	Clinician
*Tagliaferro, E.* [59]	(Cost-effectiveness analysis of preventive methods for occlusal surface according to caries risk: results of a controlled clinical trial)	2013	Brazil	CEA	Caries	DMFS/number of occlusal surfaces saved	Clinician
*Tickle, M.* [60]	A randomised controlled trial to measure the effects and costs of a dental caries prevention regime for young children attending primary care dental services	2016	UK	CEA	Caries	Conversion of teeth from caries-free to caries-active state, dmfs	Clinician
*Tonmukayakul, U.* [61]	Cost-effectiveness analysis of the atraumatic restorative treatment-based approach to managing early childhood caries	2017	Australia	CEA	Caries	Number of referrals to specialists/ number of fillings/ extractions	Clinician
*Vermaire, J.* [62]	Value for money: economic evaluation of two different caries prevention programmes compared with standard care in a randomized controlled trial	2014	Netherlands	CEA	Caries	DMFS (prevented DMFS)	Clinician
*Weintraub, J.* [63]	Treatment outcomes and costs of dental sealants among children enrolled in Medicaid	2001	USA	CEA	Caries	Caries-related services involving the occlusal surface (CRSOs)	Clinician
*Wiedel, A.* [64]	A cost minimization analysis of early correction of anterior crossbite - A randomized controlled trial	2016	Sweden	CMA	Malocclusion	Success rate of anterior crossbite correction and overjet in mm	Clinician
*Wu, Y.* [65]	(Cost-minimization analysis of two methods during the prevention of dental fear during caries filling treatments)	2002	China	CMA	Dental fear	Venhams anxiety scale	Clinician
*Yee, R.* [66]	A cost-benefit analysis of an advocacy project to fluoridate toothpastes in Nepal	2004	Nepal	CUA	Caries	DMFS	Clinician
*Zabos, G.* [67]	Cost-effectiveness analysis of a school-based dental sealant program for low-socioeconomic-status children: A practice-based report	2002	USA	CEA	Caries	DMFS	Clinician

*CEA: Cost-effectiveness analysis*

*CMA: Cost-minimisation analysis*

*CUA: Cost-utility analysis*

*CBA: Cost-benefit analysis*

Of the 46 studies included in the final analysis, three were written in languages other than English, namely Portuguese (*n* = 2, 4%) and Mandarin (*n* = 1, 2%) [29, 59, 65]. The vast majority of studies undertook cost-effectiveness analyses (*n* = 38, 82%). One study reported a cost-benefit analysis alone (2%), one study reported a cost-utility analysis alone (2%) and two studies carried out two different types of analyses (4%). Four studies (9%) reported the findings of cost-minimisation analyses.

**Fig. 2:**
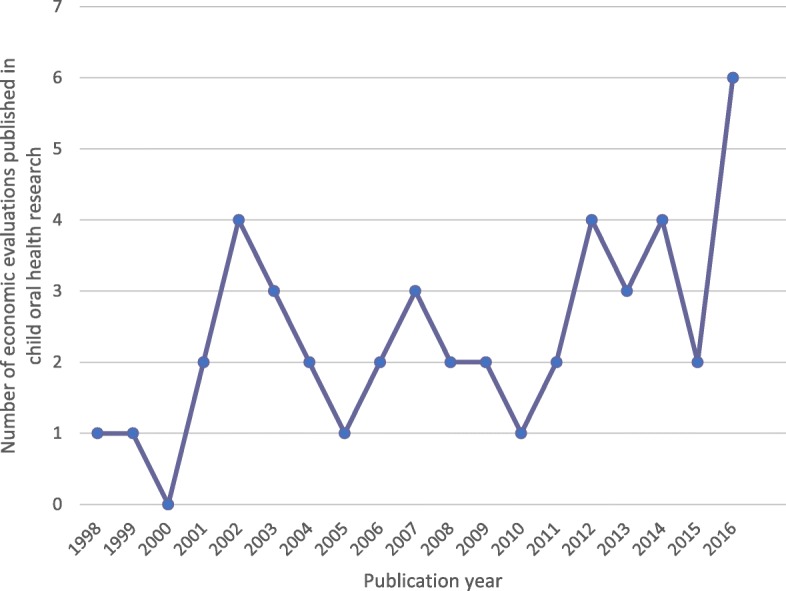
Graph displaying trends in the publication of economic evaluations in the field of child oral health research from 1998 to 2016

Figure 2 reveals the general trend for an increase in publications in this field, with an apparent peak in 2016 (*n* = 6, 13%). It should be noted that publications from 2017 have been excluded from this figure, as the review did not cover the full year.

**Fig. 3:**
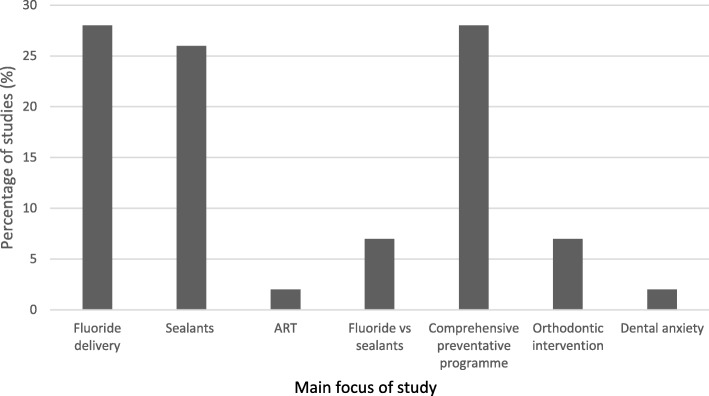
Chart displaying range of interventions provided in included studies

As displayed in Fig. 3, most studies focused on the prevention or management of dental caries (*n* = 42, 91%), with only three studies (7%) relating to malocclusion and one on dental fear (2%). The cost-effectiveness of a standard preventive programme was compared with a more comprehensive or targeted preventive programme in 13 studies (28%). No studies investigated the cost-effectiveness of interventions for MIH or traumatic dental injuries.

Outcomes were reported by clinicians in the majority of studies (*n* = 43, 87%). One study gained child-reported outcomes, which were used in combination with clinician-reported outcomes. However, this was not a validated tool and the findings did not contribute to the cost-effectiveness analysis.

As seen in Table 1, a range of outcome measures were used in the studies. Validated measures were used in 26 studies (56%), notably standard caries experience indices: dmfs/DMFS (*n* = 9, 20%), DFT (*n* = 3, 7%) and dmft/DMFT (*n* = 7, 15%). Non-validated outcome measures, such as ‘time spent on treatment’, and ‘presence of caries’ were used in 20 studies (44%). Only two studies (4%) quantified health outcomes in terms of utilities. One reported outcomes in QALYs, for which data were collected using a paediatric preference-based measure known as the CHU9D (Child Health Utility 9 Dimensions). The other study reported outcomes in Quality Adjusted Tooth Years (QATYs), a dental variation of the QALY.

The overall mean percentage of applicable Drummond checklist criteria met by the studies in this review was found to be 48%, with a range of 0 to 100% (Table 2). The median score was calculated at 50%. Two studies (4%) met all the applicable criteria, scoring 100%, whilst two studies (4%) failed to meet any of the applicable criteria, scoring 0%.

**Table 2:** Table displaying the percentage of applicable Drummond and CHEERS criteria met by each paper, with categorisation to indicate overall quality

First author	% applicable Drummond criteria met	Overall methodological quality	% applicable CHEERS criteria met	Overall reporting quality
*Alkhadra, T.* [22]	38	Moderate	65	Moderate
*Atkins, C.* [23]	46	Moderate	96	High
*Bergström, E.* [24]	23	Low	70	Moderate
*Bertrand, É.* [25]	54	High	91	High
*Bhuridej, P.* [26]	92	High	86	High
*Chi, D.* [27]	69	High	96	High
*Davies, G.* [28]	54	High	90	High
*Frazão, P.* [29]	54	High	48	Low
*Goldman, A.* [30]	85	High	90	High
*Goldman, A.* [31]	100	High	95	High
*Griffin, S.* [32]	77	High	87	High
*Hichens, L.* [33]	36	Moderate	84	High
*Hietasalo, P.* [34]	69	High	50	Low
*Hirsch, G.* [35]	0	Low	39	Low
*Holland, T.* [36]	62	High	35	Low
*Jokela, J.* [37]	8	Low	60	Low
*Kaakko, T.* [38]	15	Low	52	Low
*Koh, R.* [39]	77	High	96	High
*Kowash, M.* [40]	15	Low	85	High
*Leskinen, K.* [41]	8	Low	76	Moderate
*Marino, R.* [42]	77	High	87	High
*Mariño, R.* [43]	54	High	67	Moderate
*Mariño, R.* [44]	54	High	76	Moderate
*Morgan, M.* [45]	38	Moderate	81	Moderate
*Neidell, M.* [46]	38	Moderate	77	Moderate
*Ney, J. P.* [47]	38	Moderate	87	High
*Oscarson, N.* [48]	85	High	90	High
*Ouyang, W.* [49]	69	High	87	High
*Petrén, S.* [50]	38	Moderate	62	Low
*Pukallus, M.* [51]	54	High	100	High
*Quiñonez, R.* [52]	54	High	82	Moderate
*Quinonez, R.* [53]	62	High	95	High
*Ramos-Gomez, F.* [54]	8	Low	55	Low
*Sakuma, S.* [55]	62	High	62	Low
*Samnaliev, M.* [56]	46	Moderate	100	High
*Sköld, U.* [57]	62	High	91	High
*Stearns, S.* [58]	46	Moderate	100	High
*Tagliaferro, E.* [59]	77	High	43	Low
*Tickle, M.* [60]	38	Moderate	95	High
*Tonmukayakul, U.* [61]	46	Moderate	95	High
*Vermaire, J.* [62]	100	High	90	High
*Weintraub, J.* [63]	0	Low	78	Moderate
*Wiedel, A.* [64]	23	Low	71	Moderate
*Wu, Y.* [65]	8	Low	33	Low
*Yee, R.* [66]	23	Low	71	Moderate
*Zabos, G.* [67]	31	Low	57	Low
Categorisation	Drummond criteria met		CHEERS criteria met	
High	> 50%		> 83%	
Moderate	32–50%		63–83%	
Low	< 32%		< 63%	

The overall mean percentage of applicable CHEERS criteria met by each study was calculated at 77%, with a range of 33–100% and a median of 83% (Fig. 7). Only three studies (7%) satisfied all applicable criteria (scoring 100%).

The median percentage of Drummond and CHEERS criteria met was then used to further classify each study into high, moderate and low quality categories, to ensure that studies with a larger number of ‘not applicable’ criteria would not be unfairly disadvantaged (see legend for Table 2). A total of 23 studies (50%) were classified as high methodological quality, 11 (24%) as moderate quality, and 12 (26%) as low quality in relation to the Drummond checklist. Additionally, 23 studies (50%) were categorised as having high reporting quality, 11 (24%) as moderate quality, and 12 (26%) as low quality in relation to the CHEERS checklist. Whilst the overall number of studies in each category was the same for each checklist, it should be noted that these were not the same individual studies.

Tables 3 and 4 show how many of the included studies met each criterion from the Drummond and CHEERS checklists. A common methodological deficiency surrounded the issue of discounting; a process whereby costs and outcomes that occur in the future are adjusted to their present values. Discounting is important due to ‘time preference’ which is the desire to enjoy benefits in the present while deferring any negative effects of doing so [18, 68]. Prior to undertaking this quality appraisal, the reviewers agreed that discounting should be considered by all studies over 2 years in duration.

**Table 3:** Table displaying the total number of studies which met each criterion of the Drummond checklist

Drummond Criterion	Summary of criterion	Total studies meeting criterion n = 46 (%)		Total studies not meeting criterion *n* = 46 (%)		Total studies to which criterion is not applicable *n* = 46 (%)	
1	Was a well-defined question posed in answerable form?	26	(57)	20	(43)	0	(0)
2	Was a comprehensive description of the of the competing alternatives given?	21	(46)	25	(54)	0	(0)
3	Was there evidence that the programme’s effectiveness had been established?	27	(59)	19	(41)	0	(0)
4	Were all the important and relevant outcomes and costs for each alternative identified?	14	(30)	32	(70)	0	(0)
5a	Were outcomes measured accurately in appropriate units prior to evaluation?	31	(67)	15	(33)	0	(0)
5b	Were costs measured accurately in appropriate units prior to evaluation?	12	(26)	34	(74)	0	(0)
6a	Were the outcomes valued credibly?	12	(26)	34	(74)	0	(0)
6b	Were the costs valued credibly?	12	(26)	34	(74)	0	(0)
7a	Were outcomes adjusted for different times at which they occurred?	13	(28)	33	(72)	1	(2)
7b	Were costs adjusted for different times at which they occurred?	29	(63)	17	(37)	1	(2)
8	Was an incremental analysis of the outcomes and costs of alternatives performed?	28	(61)	18	(39)	0	(0)
9	Was allowance made for uncertainty in the estimates of costs and consequences?	32	(70)	14	(30)	0	(0)
10	Did the presentation and discussion of study results include all of the issues that are of concern to users?	30	(65)	16	(35)	0	(0)

**Table 4:** Table displaying the total number of studies which met each criterion of the CHEERS checklist

CHEERS criterion	Summary of criterion	Total studies meeting criterion n = 46 (%)		Total studies not meeting criterion n = 46 (%)		Total studies to which criterion is not applicable n = 46 (%)	
1	Title	44	(96)	2	(4)	0	(0)
2	Abstract	45	(98)	1	(2)	0	(0)
3	Background and objectives	44	(96)	2	(4)	0	(0)
4	Target population and subgroups	41	(89)	5	(11)	0	(0)
5	Setting and location	40	(87)	6	(13)	0	(0)
6	Study perspective	33	(72)	13	(28)	0	(0)
7	Comparators	41	(89)	5	(11)	0	(0)
8	Time horizon	40	(87)	6	(13)	0	(0)
9	Discount rate	29	(63)	16	(35)	1	(2)
10	Choice of health outcomes	42	(91)	4	(9)	0	(0)
11	Measurement of effectiveness	41	(89)	5	(11)	0	(0)
12	Measurement and valuation of preference-based outcomes	2	(4)	0	(0)	44	(96)
13	Estimating resources and costs	38	(83)	8	(17)	0	(0)
14	Currency, price date and conversion	36	(78)	10	(22)	0	(0)
15	Choice of model	13	(28)	5	(11)	28	(61)
16	Assumptions	15	(33)	2	(4)	29	(63)
17	Analytical methods	33	(72)	13	(28)	0	(0)
18	Study parameters	29	(63)	17	(37)	0	(0)
19	Incremental costs and outcomes	40	(87)	6	(13)	0	(0)
20	Characterising uncertainty	26	(57)	20	(43)	0	(0)
21	Characterising heterogeneity	8	(17)	18	(39)	20	(43)
22	Study findings, limitations, generalisability and current knowledge	36	(78)	10	(22)	0	(0)
23	Source of funding	31	(67)	15	(33)	0	(0)
24	Conflicts of interest	9	(20)	37	(80)	0	(0)

Within the included studies, discounting of costs was more likely to have been undertaken that discounting of outcomes, with 29 studies (64%) discounting costs and only 13 studies (29%) conducting appropriate discounting of outcomes. It also became apparent, during the review process, that a number of studies erroneously stated that discounting of costs and outcomes was not necessary for their particular study. Regarding the reporting of discounting overall, a total of 29 studies (64%) were considered to have undertaken this appropriately.

In contrast, consideration of uncertainty in the estimates of costs and consequences was found to be the best performing area, according to the Drummond criteria. A total of 32 studies (70%) conducted appropriate statistical analyses, including sensitivity analysis (where appropriate), the latter being important to assess the robustness of the conclusions drawn from an economic evaluation [18]. Nonetheless, only 26 (57%) studies were found to have reported the management of uncertainty appropriately as determined by the CHEERS checklist.

The poorest performing criterion in the CHEERS checklist related to the reporting of any conflict of interest. A total of 37 studies (80%) made no comment regarding this, thereby failing to acknowledge any potential introduction of bias.

### Rater reliability

Cohen’s kappa (κ) was calculated at 0.8 (90% agreement) for overall inter-rater agreement for the Drummond checklist, and at 0.7 (86% agreement) for the CHEERS checklist. According to the classifications proposed by Landis and Koch, these figures indicate substantial strength of agreement [69]. Furthermore, the reviewers assessed 10% of the included studies (*n* = 5) a second time to determine intra-rater reliability. The studies were selected randomly using an online random number generator. Intra-rater agreement was 94% for ZM (κ = 0.87) and 83% for HDR (κ = 0.64) using the Drummond checklist, and 88% for HJR (κ = 0.72) and 85% for EV (κ = 0.66) using the CHEERS checklist.

## Discussion

To the authors’ knowledge, this is the first systematic review to explore both the methodological quality and reporting quality of economic evaluations with respect to child oral health research. This review has highlighted a paucity of high-quality economic evaluations in this field, with a lack of active involvement of children.

The overall reporting quality of economic evaluations in the present study was relatively high, with a median score of 83% against the CHEERS criteria. However, this was less than the median score of 92% identified in a recent systematic review of economic evaluations of oral health interventions by Hettiarachchi and coworkers, which used the same CHEERS checklist [11]. The median score for overall methodological quality for the studies included in the present review was 50%, meaning that only 50% of the studies met half of the Drummond checklist criteria. The median appraisal score for full economic evaluations in the wider field of dentistry as a whole was reported to be was much higher at 85% (satisfying 11 out of a possible 13 criteria) by Tonmukayakal and coworkers [10]. This finding suggests that the methodology and reporting of economic evaluations in the narrower scope of child oral health research, is of reduced quality compared to dentistry overall. Moreover, each checklist identified 12 low quality studies, but importantly, these were not the same 12 studies. Thus there is a clear indication to employ both checklists in order to comprehensively appraise both the methodological and reporting quality of future studies in this field.

This review confirmed a lack of discounting of costs and outcomes within economic evaluations in child oral health research; an issue which was also highlighted by Tonmukayakal and coworkers within economic evaluations in dentistry overall [10]. Whilst the need to discount costs was acknowledged by most authors, some confusion was evident surrounding discounting of outcomes, which may be a reflection of the ongoing debate amongst health economists on this topic [70, 71].

The area of least compliance with acknowledged quality criteria was the reporting of any conflicts of interest, a finding also reported by Hettiarachchi and coworkers [11]. The CHEERS checklist was designed to be used for economic evaluations in the same way that the CONSORT checklist is used for quality appraisal of publications arising from trials [20]. Whilst a number of medical journals have openly endorsed the CHEERS checklist, and expect submitting authors to comply with the requirements, this does not appear to be the case for dental journals. Until these quality standards are universally applied, flaws and omissions in the reporting of dental-related economic evaluations may well continue.

Hettiarachchi and coworkers reported an increase in the publication of cost-utility analyses in dentistry over recent years [11]. However, this trend was not reflected in the present review with only two studies using this approach. One measured utilities using the QALY (Quality Adjusted Life Years), a measure of health benefit that combines both quality of life and length of life into a single index. In order to measure this quality of life, a preference based measure is needed with a weighting assigned to each health state defined by the descriptive system, on a 1–0, full health to dead scale. As mentioned above, the study in question used the CHU9D, a generic paediatric multi-attribute instrument, which was developed with involvement of children and young people [12]. Unfortunately, research indicates the CHU9D to be unresponsive to the changing components of dental caries experience, which may limit the applicability of this measure to child oral health research [72].

The other study with a cost-utility approach used the lesser-known Quality Adjusted Tooth Year (QATY). The QATY was developed as a dental-variation of the QALY [73–75], yet its use within the literature has been minimal due to a number of limitations. Notably, the QATY cannot be used for all dental interventions, and has limitations when used in relation to the primary dentition. Furthermore, it does not take account of the strong and important link between oral health and general health. Acknowledging this, the QALY remains the primary means of representing strength of preference as advocated by NICE. Nonetheless, there is a clear need for the development of a paediatric preference-based measure of dental caries to facilitate greater use of the QALY in future economic analyses.

The overwhelming majority of studies in the present review conducted a cost-effectiveness analysis (*n* = 38, 83%). A range of outcome measures were employed, most being a variation of the DMFT index (Decayed, Missing and Filled Teeth), which has been widely used for over half a century as a means of collecting easily comparable data on caries prevalence and treatment provision from different populations [76]. Unfortunately, there are so many variations stemming from this index alone, such as the DMFS (Decayed, Missing and Filled Surfaces), DFS (Decayed and Filled Surfaces), DFT (Decayed and Filled Teeth) indices, that meaningful comparisons between studies are complex. The use of additional outcome measures, such as ‘number of caries-free teeth’, ‘number of caries averted’ and ‘number of caries-free months’, further precludes inter-study comparisons, preventing data from being maximised through systematic reviews, and ultimately disrupting the dissemination of study findings across the world.

Difficulties arising from the use of so many different outcome measures in economic evaluations has not gone unnoticed, and has been highlighted by authors of previous systematic reviews [77, 78]. This has led to the initiation of the Outcomes in Trials for Management of Caries Lesions (OuTMaC) study, which aims to develop a core outcome set for trials investigating management of caries lesions in primary or permanent teeth [78]. This study is currently in progress, though it intends to use Delphi methods to facilitate panel agreement for a maximum of seven outcome measures for use in this field. It is anticipated that the findings from this study will ultimately improve the measurement of benefits in economic evaluations within child oral health research.

Lack of meaningful involvement of children was a key flaw within the included studies. No single study considered children’s perspectives, potentially overlooking issues relating to oral health which would be of direct relevance to children themselves. The importance of involvement of children in both research and healthcare decisions is increasingly acknowledged, hence there is scope for substantial improvement within future economic evaluations. One way to accomplish this would be to gain preferences from children in the development of a dental utility measure. Whilst this methodology is not yet widely used in healthcare, research indicates that it is both feasible and reliable [79].

### Strengths and limitations

A particular strength of the present study was the involvement of a multidisciplinary team, bringing expertise from a number of different dental specialties and health economics. Whilst this study did not apply any language restrictions, the studies published in languages other than English were only reviewed by one calibrated reviewer (HJR), working alongside a translator. The translators used were native language speakers, and either dentists or health economists, so the terminology used within the studies was familiar to them.

An acknowledged limitation of this study, however, was the exclusion of modelling studies which included young people over the age of 18-years. This review intended to focus on studies which explored the benefits of interventions gained, and associated costs incurred, solely during childhood. Nonetheless, these modelling studies play an important role in acknowledging that oral health interventions administered during childhood can have benefits (and associated costs) that extend far beyond childhood.

### Areas for future research

It is clear from the focus of studies included in this review that prevention and management of dental caries remains at the forefront of the global paediatric oral health agenda. However, it was surprising that economic evaluations relating to other common, and potentially burdensome, childhood dental conditions were sparse. It is proposed that MIH and traumatic dental injuries are conditions which also present considerable societal and healthcare impacts, and thus should be priorities for future economic research.

Most importantly, this review has identified the need to develop a dental child-centred and preference-based measure to address the need to involve children in research, and to provide a suitable instrument for determining QALY data.

## Conclusion

There is a paucity of high-quality economic evaluations in the field of child oral health. This deficiency could be addressed through the endorsement of economic evaluation guidelines by dental journals. The development of a utility measure for use in paediatric oral health research would also seem to be indicated to facilitate children’s engagement in future economic evaluations of dental conditions and interventions.
